# Treatment and outcome in medication overuse headache patients

**DOI:** 10.1186/1129-2377-14-S1-P180

**Published:** 2013-02-21

**Authors:** D Mitsikostas, I Deligianni

**Affiliations:** 1 Athens Naval Hospital, Greece

## Background

Medication overuse headache (MOH) is a frequent condition in headache centers with elusive management and outcomes. Aim: to present the pharmaceutical treatment and outcome of MOH patients from the outpatient Headache Clinic of the Athens Naval Hospital.

## Methods

This is an open, retrospective, single center observational study. The electronic files of patients with MOH of the Athens Naval Hospital Out-Patient Headache Clinic were reviewed to assess the outcome.

## Results

One hundred forty six patients (29 males and 117 females) with MOH were evaluated (mean age 38.5 ±12.7 years; mean body mass index 26.1±4.44). Patients overused triptans (38.9%), analgesics (61.1%), NDAIDs (48.4%), codein plus paracetamol (77.6%), bezodiazepines (2.3%), or ergotamine (17.5%). Scores for Hamilton scale for anxiety and depression were 23.9±5.6 and 18.8±6.5, respectively. Prophylactic treatment consisted of naproxen 1000mg (plus gastroproplylaxis), SNRI (venlafaxine 150-300mg/d, or mirtazapine 45-90mg/d) and one of three agents: propranolol (160-320mg/d), topiramate (100mg) or valproate (500-1500mg/d). In cases of anxiety disorder comorbidity (HAM-A score>18) prazepam was added (10-20mg/d). All patients were advised to withdraw immediately the substance overused. After one to three months treatment 37% and 45.6% of patients reported a greater of 50% or 75% decrease of days with headache per month, respectively. The 17.3% of the patient population did not respond to treatment.

## Conclusion

Immediate withdraw of substance overused combined with a strong prophylactic treatment, which included naproxen, SNRIs and preventative antimigraine agents is efficient to decrease headache impact in most patients with MOH.

